# The Discovery of Potent SHP2 Inhibitors with Anti-Proliferative Activity in Breast Cancer Cell Lines

**DOI:** 10.3390/ijms23084468

**Published:** 2022-04-18

**Authors:** Rose Ghemrawi, Mostafa Khair, Shaima Hasan, Raghad Aldulaymi, Shaikha S. AlNeyadi, Noor Atatreh, Mohammad A. Ghattas

**Affiliations:** 1 College of Pharmacy, Al Ain University, Abu Dhabi 112612, United Arab Emirates; rose.ghemrawi@aau.ac.ae (R.G.); shaima.hasan@aau.ac.ae (S.H.); 2 AAU Health and Biomedical Research Center, Al Ain University, Abu Dhabi 112612, United Arab Emirates; raghad.aldulaymi@aau.ac.ae; 3 Core Technology Platforms, New York University Abu Dhabi, Abu Dhabi 129188, United Arab Emirates; mrk6@nyu.edu; 4 Department of Chemistry, College of Science, UAE University Al-Ain, Abu Dhabi 15551, United Arab Emirates; shaikha.alneyadi@uaeu.ac.ae

**Keywords:** breast cancer, protein tyrosine phosphatase SHP2, enzyme inhibitors

## Abstract

Despite available treatments, breast cancer is the leading cause of cancer-related death. Knowing that the tyrosine phosphatase SHP2 is a regulator in tumorigenesis, developing inhibitors of SHP2 in breast cells is crucial. Our study investigated the effects of new compounds, purchased from NSC, on the phosphatase activity of SHP2 and the modulation of breast cancer cell lines’ proliferation and viability. A combined ligand-based and structure-based virtual screening protocol was validated, then performed, against SHP2 active site. Top ranked compounds were tested via SHP2 enzymatic assay, followed by measuring IC50 values. Subsequently, hits were tested for their anti-breast cancer viability and proliferative activity. Our experiments identified three compounds **13030**, **24198**, and **57774** as SHP2 inhibitors, with IC50 values in micromolar levels and considerable selectivity over the analogous enzyme SHP1. Long MD simulations of 500 ns showed a very promising binding mode in the SHP2 catalytic pocket. Furthermore, these compounds significantly reduced MCF-7 breast cancer cells’ proliferation and viability. Interestingly, two of our hits can have acridine or phenoxazine cyclic system known to intercalate in ds DNA. Therefore, our novel approach led to the discovery of SHP2 inhibitors, which could act as a starting point in the future for clinically useful anticancer agents.

## 1. Introduction

Breast cancer is the leading cause of cancer-related death among women worldwide [1]. According to WHO, the number of cases will increase, and cases are expected to double by 2030 [2]. The suboptimal treatment outcomes despite chemotherapy and immunotherapy show that enzymes’ targeted therapies are urgently needed to treat breast cancer cases.

The coordinated function of protein tyrosine kinases and protein tyrosine phosphatases (PTPs) maintain the levels of tyrosine phosphorylation, which is known to be critical for a wide range of cellular processes, such as growth, differentiation, metabolism, migration, and survival [3,4]. The deregulation of tyrosine phosphorylation was linked to many types of cancer, especially breast cancer [5].

Therefore, signaling pathways regulated by tyrosine phosphorylation may offer novel molecular targets for therapeutic interventions [6,7,8,9].

Src homology region 2 protein tyrosine phosphatase 2 (SHP2), encoded by PTPN11, is the first reported non-receptor protein oncogenic tyrosine phosphatase and is required for the survival, proliferation, and differentiation of multiple cell types [10]. It plays a regulatory role in signal transduction downstream of multiple receptor tyrosine kinases, such as PI3K/AKT, RAS/RAF/MEK/ERK, and RAS/MAPK signaling [11,12,13]. A mutation of SHP2 leading to a gain in its function was correlated with breast tumorigenesis and cancer progression [14,15]. Therefore, the development of small-molecule inhibitors of SHP2 may provide the opportunity to inhibit oncogenic signaling in breast cells. Many synthetic compounds have shown an inhibitory effect on SHP2 [16], by targeting the catalytic site (e.g., thiazolidinone derivatives and NSC 87877) [17] or the allosteric site (e.g., SHP099) [18]. Furthermore, SHP2 inhibition and the combination of SHP2 and MEK or ERK inhibitors, were recently found to enhance antitumor efficacy in different types of cancer [16]. 

In the present study, we investigated the effects of new compounds on the phosphatase activity of SHP2 and the modulation of breast cancer cell lines’ proliferation and viability. A structure-based virtual screening against the SHP2 active site was performed based on a pre-validated computational approach. Top ranked compounds were biologically tested via SHP2 enzyme assay to confirm their binding with the target protein. Then, the IC50 concentrations were calculated. Subsequently, these hits were tested for their anti-breast cancer viability and proliferative activity.

## 2. Results and Discussion

### 2.1. Pharmacophore Model Generation and Validation

The goal of employing pharmacophoric-filters in virtual screening (VS) is to find interesting ligands that could exert inhibitory activity against SHP2 while significantly minimizing the VS time. This approach has been employed in many studies and has always shown superior performance over the standard VS protocol [19,20]. Hence, in this study, we validated the suitability of the pharmacophoric filter-based VS for the discovery of new SHP2 inhibitors. Consequently, the said protocol was employed via screening the National Cancer Institute (NCI) ligand library against the SHP2 active site, where top hits were experimentally tested for their enzymatic inhibitory effect as well as for their anticancer activity using several cancer cell-lines.

Pharmacophore queries were generated based on known SHP2 active site inhibitors. A training set of five compounds (Supplementary Information Scheme S1) was prepared for pharmacophore queries generation. These molecules have the fewest number of rotatable bonds, a varied molecular size and IC50 values, and they belong to various types of scaffolds.

Many pharmacophore queries were created from the training set compounds overlaying. Table 1 shows pharmacophore queries with the best selectivity (Se) and specificity (Sp) rates towards SHP2 active site inhibitors. Query no. 4 was the best pharmacophore model in selecting active site inhibitors over decoys, with a selectivity rate of 100% along with its distinguished ability to exclude most of the true negatives (Sp = 90%, Table 1). Only query no. 7 performed better than query no. 4 in excluding decoys, with the highest specificity rate of 93.3%; yet its selectivity rate was not the best (Se = 93.2%), as higher rates were observed from other queries (i.e., query no. 4, 39 and 42). Taken all together, query no. 4 seemed to have the edge over other queries and, hence, was selected to be used in the validation process of our VS protocol.

**Figure 1:**
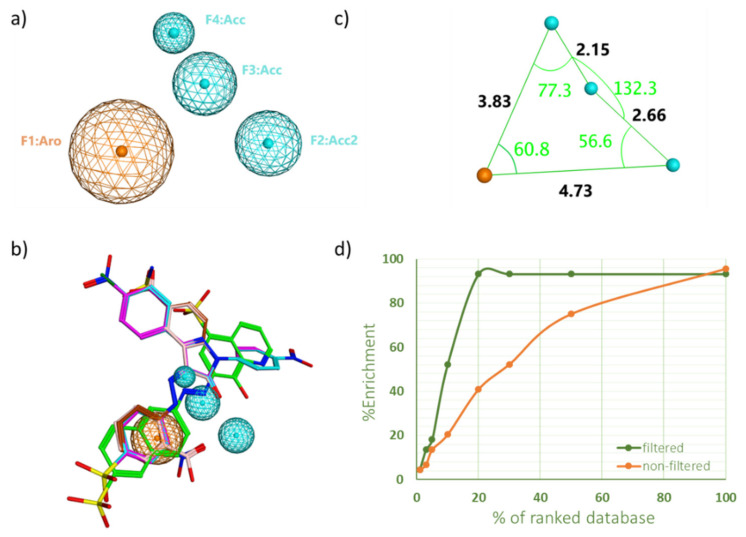
(**a**) The four features of the top pharmacophore query no. 4. (**b**) Two known inhibitors overlaid on the said pharmacophore. (**c**) The measured distances and angles between the four pharmacoohoric features (**d**) The effect of using pharmacophore query no. 4 as pre-docking filter in a pilot showing the number of known SHP2 inhibitors retrieved (%Enrichment) at any given percentage of the top-ranked ligand library.

The four features of the pharmacophore query no. 4 are shown in Figure 1a. The pharmacophore consists of one aromatic center, two hydrogen bond acceptors, and one hydrogen bond acceptor projection. Distances and angles between those features were also determined (Figure 1c). Two known inhibitors that successfully passed through the pharmacophore were overlaid on the pharmacophoric query and shown in Figure 1b. It is worth mentioning that a similar recent study has constructed a pharmacophore model for the SHP2 inhibitors, using a different pharmacophore elucidation tool (i.e., Accelry Discovery Studio 3.5 software [21]). The generated pharmacophore in that study had two hydrogen bond acceptors, one aromatic center, and two hydrophobic centers. Additional comparison to another PTP member can be found in a PTP1B study, where the resultant pharmacophore model had two hydrogen bond acceptors, two hydrophobic centers, and a negative ionic center feature. To sum up, the detected features in our pharmacophore correlate well with the PTP pharmacophores found in the literature, particularly the aromatic center and hydrogen-bond acceptors features, which meets the important two features in the natural substrate too (i.e., the phosphate group attached to an aromatic ring).

The pharmacophore query no. 4 was then tested in a seeding experiment (i.e., a pilot virtual screening experiment) in order to check if it can be used as prior docking in a conventional VS approach. To do this, a 5000-ligand library consisting of decoys and SHP2 active site inhibitors was created. We employed two virtual screening protocols: standard virtual screening (SVS) and filter-based virtual screening (FVS), where the latter protocol applies a pharmacophoric filter before docking. The % EF was calculated to determine the efficiency of each protocol in retrieving SHP2 inhibitors over decoys among the top-ranked ligands derived from virtual screening.

Table 2 compares enrichment factor values for both protocols at different portions of ranked docked ligands. Although both protocols have an equal %EF of 4.5 in the top 1% portion, FVS showed a superior performance in the rest of the screening portions, remarkably scoring more than double enrichment in the top 3% (%EF = 13.6 vs. 6.8 in SVS, and %EF = 52.27 vs. 20.5 in SVS). Additionally, FVS was able to retrieve almost all inhibitors in the top 20% portion of the VS, compared to only 40% that was scored by SVS. Overall, the % EF over various portions of the screen indicated that the FVS approach outperformed the standard protocol (Figure 1d).

### 2.2. Virtual Screening and Compounds Selection

**Figure 2:**
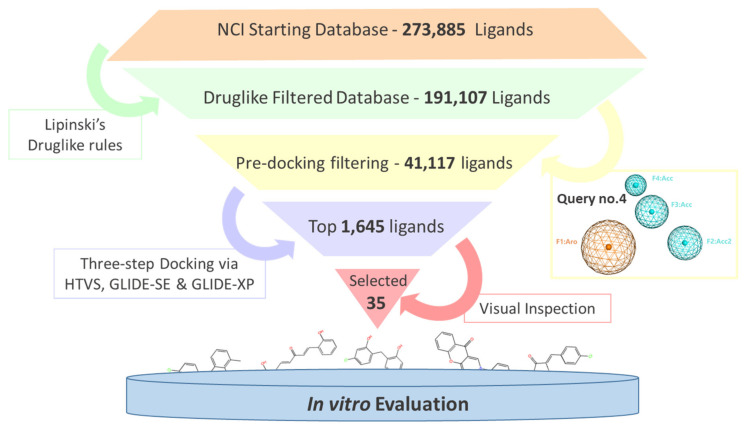
The workflow of the filter-based VS used against the SHP2 active site.

VS was conducted to screen the NCI ligand library against the SHP2 active site using our validated FVS protocol. As shown in Figure 2, the whole screening started with filtering the ligand dataset based on the druglike rules, then it was screened through the previously prepared SHP2 inhibitor pharmacophore. The druglike filtering downsized the ligand library from 273,885 ligands to 191,929 ligands, while the pharmacophore filtering had the biggest effect on the size of the library, significantly reducing its size by more than 4-fold (i.e., 41,117 ligands remained). Hence, the use of pharmacophore as pre-docking filter in VS seems to have a very positive effect not only on the quality of the results (i.e., enrichment factors), but also on saving our computational resources and speeding up the overall process [19,22].

Consequently, the final ligand dataset was docked into the SHP2 active site in a three-step process, where precision and docking accuracy increased with time. These three steps were conducted via the HTVS, SP, and XP mode in the GLIDE docking software. The final listed was ranked based on the GLID-XP scoring function and then visually inspected for their fitting and interaction with the SHP2 key residues.

### 2.3. Inhibition of SHP2 Enzyme Activity by the Compounds NSC ***13030***, ***24198***, ***57774***, and ***137420***

**Figure 3:**
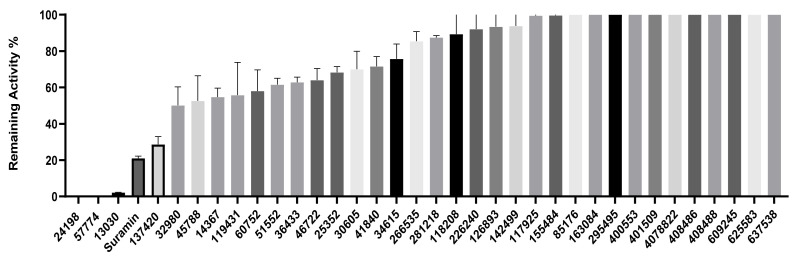
Remaining activity (%) of the SHP2 enzyme after treatment with the 35 tested compounds (at 100 μM) along with the control suramin. Data presented are the mean +/− SEM of three independent experiments.

The validation of the docking results was first performed by an enzymatic assay. Based on the ligand-SHP2 interaction profiles and the fitting into the catalytic pocket of the target enzyme, 35 compounds were selected from the NCI ligand library (National Cancer Institute Repository, Bethesda, MD, USA) and purchased. These compounds were screened initially against their inhibitory effect on SHP2 enzyme. The colorimetric enzymatic testing was carried out through an in-house validated assay. Amongst the 35 tested compounds, four hits (i.e., NSC **13030**, **24198**, **57774**, and **137420**) showed good inhibition for SHP2 enzyme at 100 μM (Figure 3). The results of inhibitory potentials of these four compounds are shown in Figure 3. The addition of the four hits compounds significantly reduced SHP2 activity (*p* < 0.05) toward its substrate, showing that they might be masking SHP2′s active site. Interestingly, **57774** showed complete inhibition, **13030**, **24198**, and **137420** exhibited 98%, 99%, and 71% inhibition, respectively. Suramin (a known competitive inhibitor of PTPs) was used as a positive control, and it showed 79% inhibition at 100 μM.

Further screening was performed on these four hits to determine their IC50. As shown in Table 3, while **137420** exhibited an IC50 value of 33 μM, the other three compounds showed low IC50 values (3.2 μM for **13030**, 1.9 μM for **24198**, and 0.8 μM for **57774**). Interestingly, these values were even lower than the IC50 of the positive control, suramin (IC50 = 4.5 μM), showing that these compounds were more potent inhibitors than suramin.

SHP2 has an analogue protein: SHP1. Both proteins have similar structure, two tandem Src homology 2 domains at the N terminus, a single central catalytic domain, and a C-terminal domain [22]. Therefore, the selectivity of these compounds for SHP2 was investigated. As shown in Table 3, three compounds, and especially **57774**, are selective for SHP2, since the obtained IC50 values with SHP1 were very high (85.4 μM for **13030**, 14.3 μM for **24198**, and 164.4 μM for **57774**) in comparison with the ones obtained with SHP2.

In conclusion, the three compounds, **13030**, **24198**, and **57774** showed good IC50 values and selectivity for SHP2. In particular, **57774** showed the best potency, with an IC50 value in the sub-micromolar level (0.8 μM) and the best selectivity with 200-fold inhibition activity towards SHP2 over its analogous enzyme, SHP1 (IC50 = 164.4 μM).

### 2.4. Compounds Inhibit Breast Cancer Cells’ Proliferation and Viability

**Figure 4:**
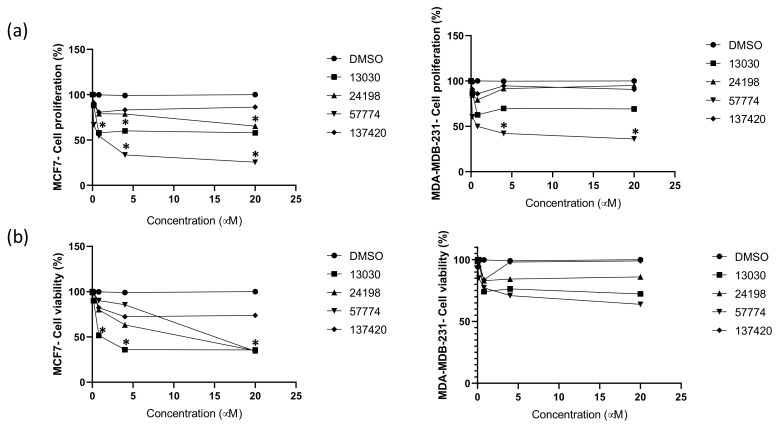
The effect of the compounds NSC **13030**, **24198**, **57774**, and **137420** on MCF-7 and MDA-MB-231 proliferation (**a**) and viability (**b**). Cells were incubated with increasing concentrations of compounds in culture medium for 48 h. The viability and the proliferative response were assessed. Data presented are the mean ± SEM of three independent experiments. * *p* < 0.05. Continuous lines are for compounds, and dashed lines are for the vehicle DMSO.

SHP2 has been previously proposed as an important regulator in breast tumor progression with therapeutic potential [16]. Inhibiting SHP2 in breast cancer was correlated with extending the survival of tumor-bearing mice and suppressing cell proliferation [23]. Therefore, the proliferation inhibitory effects of **13030**, **24198**, **57774**, and **137420** compounds on two breast cancer cell lines, MCF-7 and MDA-MB-231, were measured. Interestingly, all compounds showed anticancer activity against at least one type of cell lines. While **13030**, **24198**, and **57774** significantly reduced MCF-7 proliferation in a dose dependent manner (Figure 4a), only **57774** reduced MDA-MB-231 proliferation (Figure 4a). At 20 μM, in comparison with the vehicle, **57774** reduced MCF-7 proliferation by 74.5% and MDA-MB-231 by 64%, **13030** induced a 42.1% reduction of MCF-7 proliferation, and **24198** reduced it by 34.7%. These results were in concordance with cells’ viability. As shown in Figure 4b, while at 20 μM, compounds **13030** and **24198** reduced the viability of MCF-7 by 64.4% and 65.2%, respectively, **57774** had an effect on both MCF-7 and MDA-MB-231 cell lines. At 20 μM of **57774**, MCF-7 viability was reduced by 65.8% and MDA-MB-231 by 36.2%. It is worth noting that MDA-MB-231 is a triple-negative (TNBC), and MCF-7 is an estrogen (ER) positive breast cancer cell line. While finding potential novel solutions to treat ER positive breast cancer is easier and shows a favorable outcome, treating TNBC has limited therapeutic approaches [24]. Interestingly, here, in addition to the compounds which reduced MCF-7 proliferation and viability, we found novel compound **57774** to be able to inhibit not only MCF-7 but also MDA-MB-231 proliferation and viability.

### 2.5. Molecular Dynamic Studies for Compounds Stability and Fitting inside the SHP2 Active Site

**Figure 5:**
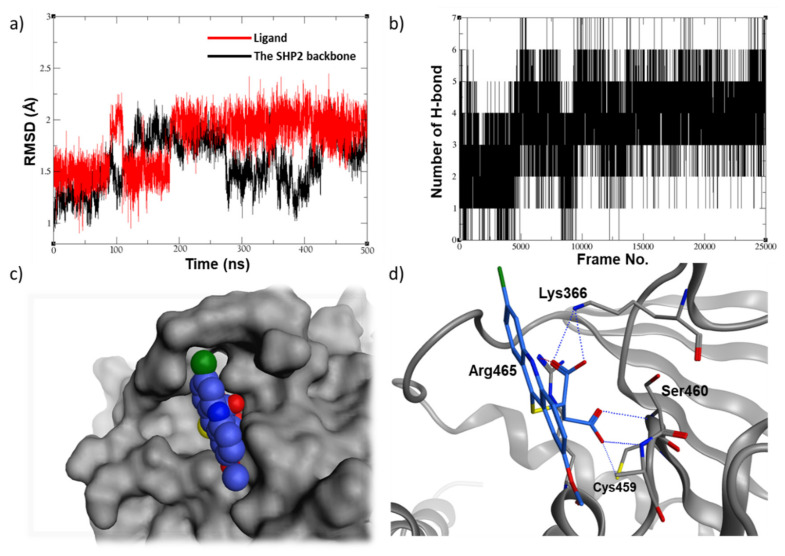
(**a**) The RMSD plot of the 500 ns MD simulations of compound **13030** along with (**b**) the hydrogen bonding plot, (**c**) the top-cluster conformation filling (blue spheres) in the SHP2 active site (gray surface), and (**d**) the ligand 3D binding mode showing the key interactions made with the surrounding residues.

For a comprehensive understanding of protein-ligand binding mode, a 500 ns molecular dynamic simulation was conducted for the three top ligands that showed a promising inhibitory activity and selectivity. As shown in Figure 5a, the acridine-based compound, **13030**, had its RMDS values converged just after the 200 ns point, oscillating around the 2 Å value. It seems that this compound had changed conformation by the 200-ns step as it was searching for a better binding mode, which is evident by the increase of the number of hydrogen bonding which increased from the range of 2–4 (0 to 200 ns) to the range of 4–6 hydrogen bonds (200 ns to the rest of the simulation) (Figure 5b). Furthermore, **13030** seems to have a good fitting in the SHP2 active site (Figure 5c) and appears to form several ionic and hydrogen bonds with the surrounding residues, including the key residue, Arg465 (Figure 5d).

**Figure 6:**
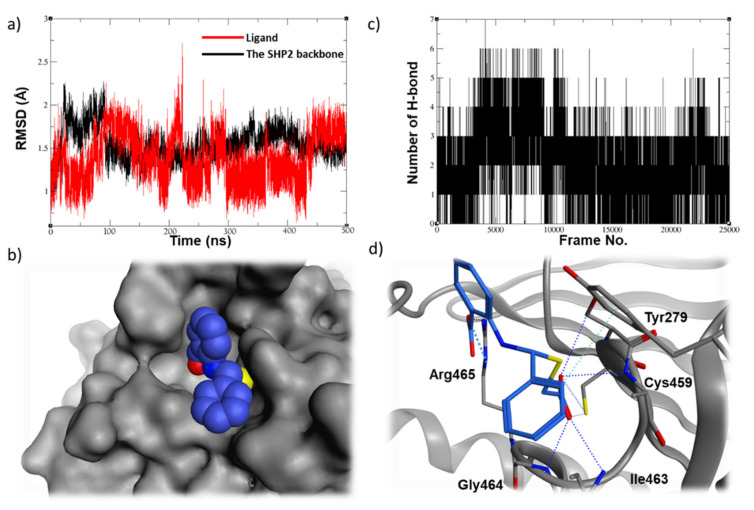
(**a**) The RMSD plot of the 500 ns MD simulations of compound **24198** along with (**b**) the hydrogen bonding plot, (**c**) the top-cluster conformation filling (blue spheres) in the SHP2 active site (gray surface), and (**d**) the ligand 3D binding mode showing the key interactions made with the surrounding residues.

Compared to the above hit, **24198** seems too to be stable in the SHP2 active site (Figure 6a), with a similar ability of hydrogen bonding throughout the course of the simulation (4–6 hydrogen bonds, Figure 6b). The ligands fill in the binding site nicely (Figure 6c) and appear to form several hydrogen bonds by its first carboxylate, particularly with the backbone amide of the P-loop, and to make an important ionic interaction with the key residue Arg465 by its second carboxylate (Figure 6d).

**Figure 7:**
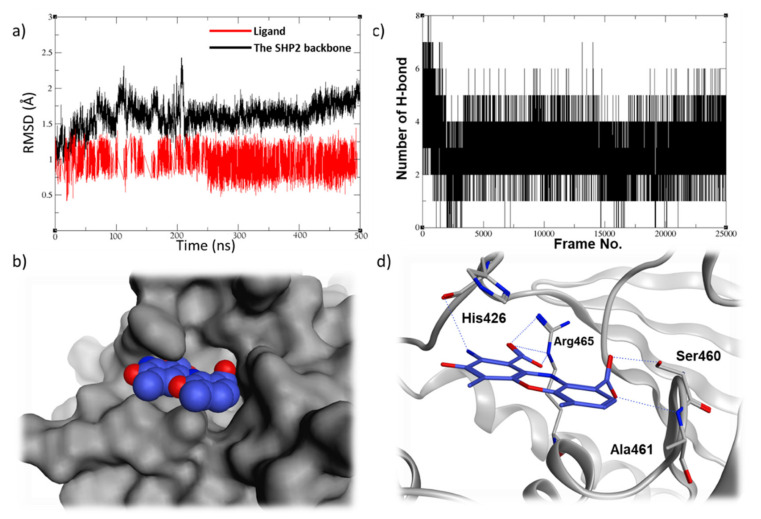
(**a**) The RMSD plot of the 500 ns MD simulations of compound **57774** along with (**b**) the hydrogen bonding plot, (**c**) the top-cluster conformation filling (blue spheres) in the SHP2 active site (gray surface), and (**d**) the ligand 3D binding mode showing the key interactions made with the surrounding residues.

Finally, the phenoxazine-based compound, **57774**, was able to demonstrate a very stable binding mode in the SHP2 pocket, being able to keep the RMSD values oscillating around 1Å over the course of the MD simulation (Figure 7a). It was able to keep at least four hydrogen bondings with the SHP2 residues, which correlates very well with the low RMSD values observed for this ligand (Figure 7b). Furthermore, it exhibited a very promising fitting and interaction profile, being able to have a very good filling to the active site (Figure 7c) and to form several ionic and hydrogen bonds with the P-loop residues Ser460, Ala461, and Arg465, along with the WPD loop residue His426 (Figure 7d). The MD simulation data of the compound **57774** came in line with its very promising in-vitro enzymatic inhibition and anti-proliferative data. Although this hit has two carboxylate groups that could hinder its cell permeability and oral activity, **57774** can still be viewed as an interesting starting point as it has a small size and lead-like characteristics, providing big room for future development. For instance, these carboxylate groups can be replaced with a more viable isostere, such as tetrazole, which has less acidic characteristics and, hence, better pharmacokinetic profile A [25,26].

It is worth mentioning that compound **13030** belongs to the acridines family which usually demonstrates antitumor activity, particularly as intercalating agents. Due to the planarity present in their tricyclic system, acridines are able to slide in between the DNA nitrogen bases, and block the DNA replication and transcription processes [27,28,29]. Similarly, compound **57774** has a phenoxazine ring system in its structure which was experimentally proven to bind to DNA and exert anticancer effect [30,31]. It was suggested by docking that such compounds are able to interfere with the DNA vital processes either by an intercalating mechanism or through binding to the DNA minor groove [32]. Hence, our compounds **13030** and **57774** might possess dual anticancer effect, possibly through acting as SHP2 inhibitors synergized by their potential activity as intercalating agents and leading to their remarkable antiproliferative activity, previously shown in our cell-based assays (Figure 4).

### 2.6. Assessment of Pharmacokinetic and Druglike Characteristics

Our four compounds were assessed via the SWISSADME [33] software for their pharmacokinetic and druglike characteristics. As shown in Table 4, all of our compounds were predicted to have high GI absorption, except **57774**, whereas none of them was predicted to have the ability to pass through the blood brain barrier (BBB). Speaking about their metabolic profile, only compound **13030** was predicted to have potential interference with the cytochrome P450 enzymes, which means that it has to be further investigated in the future for any potential drug–drug interactions. On other hand, all four compounds appeared to enjoy very satisfactory druglike properties based on many previously described rules (i.e., Lipinski’s [34], Veber’s [35], Ghose’s [36], Egan’s [37], and Mugge’s [38] rules). Moreover, none of our top hits were predicted as PAINS [39], which suggests their innocence of having potential pan assay interferences, or being frequent hitters or promiscuous compounds (aggregates are not the inhibitory species [40]). As per Brenk’s [41] guidelines for leadlike compounds, three of our compounds (**13030**, **24198** and **57774**) appeared to bear unwanted groups in their structures, such as polycyclic aromatic hydrocarbon, and aniline or hetero-carbon-hetero groups. Although these functional groups can be seen in many true inhibitors and drugs (such as the anticancer agent camptothecine and many other intercalating agents), future development and optimization of these compounds should consider replacing these potentially toxic or reactive groups with safer substitutions. All in all, our top hits seem to have satisfactory pharmacokinetic properties and druglike characteristics, which can introduce them as good candidates for future development of SHP2 inhibitor and anticancer agents.

## 3. Materials and Methods

### 3.1. Literature Review

Upon extensive literature review for SHP2 inhibitors, two set of libraries were identified and prepared; one consists of 49 active site inhibitors, and the other consists of 30 allosteric site inhibitors (to be used as true negatives). A 3D structure for all ligands in both libraries were constructed using MOE software [42]. Both databases were subjected for wash function by MOE, where all possible protonation states were generated [43].

### 3.2. Pharmacophore Generation and Validation

As a starting point, five compounds of active site inhibitors of various scaffolds, molecular weights, IC50 values, and with the least number of rotatable bonds were picked as a training set for pharmacophore generation. Pharmacophore elucidator of MOE software was used for pharmacophore generation by bringing out different overlaying sets of the superimposed conformations of the training set that sorted based on overlaying score. A set of pharmacophore features were chosen for queries generation, represented by aromatic center (Aro), H-bond donor (Don), hydrophobic centroid (Hyd), H-bond acceptor (Acc), H-bond acceptor projection (Acc2), and H-bond donor projection (Don2). Pharmacophore elucidator created a large number of queries, where the highest 50 scored queries were selected for selectivity (Se) and specificity (Sp) assessment. Selectivity evaluates the efficiency of the suggested pharmacophores in picking ligands of interest in the presence of other undesired ligands, while specificity measures pharmacophore efficiency in excluding those ligands that bind to the allosteric site [44]. The testing sets used in the pharmacophore assessment comprises 44 known SHP2 active site inhibitors and 30 allosteric site inhibitors, where the former set was considered as true positives and the latter set was employed as true negative.

### 3.3. Validation of the Virtual Screening Protocol

A library consisting of 5000 compounds was created, 44 of which were known SHP2 inhibitors, and the remaining compounds were decoys obtained from a commercial ligand library. To appraise the effectiveness of the proposed pharmacophore, the created library was used to test pharmacophore’s capability to choose inhibitors over decoys during virtual screening.

A co-crystallized structure of SHP2 with an inhibitor was downloaded from protein data bank RCSB (PDB ID: 4RDD) [45]. The protein was stripped of all water molecules and then subjected to the protein preparation wizard in the MOE software, in order to correct the protein structure for any missing atoms, residues, and loops. The structure was then processed by the Maestro software [46] for further protein adjustments, by setting up partial charges for atoms and protonation states for ionizable groups. The docking site was defined as a grid box via the GRIDE module in Maestro, considering the bounded inhibitor as a centroid of the box [47].

Energy minimized 3D structures of all possible conformations and tautomeric forms of library’s compounds were generated by LigPrep module of Maestro [48]. For the purpose of protocol validation, two virtual screening protocols were applied for the prepared ligands: a filter-based virtual screening (FVS) and a standard virtual screening (SVS). In both protocols, the previously prepared library was docked into the SHP2 active site using the standard precision mode GLIDE-SP [49], which was also used to score and rank the top ligands. However, in the FVS protocol, the generated pharmacophore was used as filter before the docking step while, in the standard protocol, docking was carried out directly with no pre-filter step. Subsequently, the enrichment factors were calculated for the two protocols to evaluate their efficiency in picking the correct active ligands. EF for the top n% was calculated using the following equation:

(1) $$EF_{top\;n\%}=\frac{number\;of\;inhibitors\;ranked\;in\;the\;top\;n\%\;of\;screened\;library}{total\;number\;of\;known\;inhibitors}\times 100\%$$

### 3.4. Virtual Screening

The ligand database of National Cancer Institute/USA NCI was employed in the validated filter-based virtual screening protocol. Thereafter, the ligand library was filtered for their drug-like characteristics based on Veber’s rules [35] and Lipinski’s rules of five [34]. Utilizing LigPrep module in Maestro, protonation states and tautomer forms of ligands were generated. In line with our approach, the prepared database was proceeded for one more filtration step where the created pharmacophore was used to eliminate any ligand that is less likely to act as a SHP2 inhibitor.

A three-steps screening protocol was utilized to select potential lead compounds [49]. To begin with, all ligands in the filtered database was docked into the previously prepared SHP2 active site via the high HTVS mode in GLIDE. For more accurate and precise docking results, the highest 20% scored ligands (8221) were re-docked using GLIDE-standard precision (SP). Lastly, a third docking step was applied for the top 20% SP docked ligands using the extra-precision algorithm (GLIDE-XP) [50]. As a result, the database was reduced to 1645 ligands, where a total of 35 compounds were visually inspected and selected by for enzymatic assay.

### 3.5. Molecular Dynamic (MD) Simulations

The crystal structure of SHP2 (PDB ID: 4RDD) was prepared using pdb4amber, where all water molecules and bounded ligands were taken off using the ff19SB force field. The best-docked conformation of the selected compounds was picked and proceeded for MD simulations. Ligands were prepared through the Antechamber program [51], applying the Generalized Amber Force Field (GAFF) and AM1-BCC [52]. Protein-ligand system was constructed using Xleap program of Amber tool. System’s charge was neutralized by Na+ counter ions and then soaked in a truncated octahedral box of TIP3P water that was 14 Å from the border.

Utilizing the pmemd program from the AMBER 18 [53], starting with a restrained complex, the system was energy minimized with a force constant of 500 kcal mol^−1^ Å^−2^, followed by minimizing the whole system without restrains. Applying molecular dynamics simulation, the energy-minimized system was then heated under NVT condition to the desired temperature of 300 K with a 10 kcal mol^−1^ Å^−2^ restraint on ligand atoms over 20 ps. The SHAKE algorithm was applied to all bonds, including hydrogen atoms, using the Langevin thermostat with a collision frequency of 1.0 ps-1. Finally, a production MD run of 500 ns for all selected compounds was carried out under NPT conditions with a target temperature of 300 K and pressure of 1 atm. Coordinates were recorded every 2 ps throughout the trajectory. We calculated binding energy using MM-GBPSA scoring [54].

### 3.6. Clustering Analysis

To find out which residues predominantly interact with ligands over the 500 ns simulation, a clustering job was run using DBSCAN by Amber’s cpptraj module for each complex [55]. Through the clustering process of the resultant MD simulation frames, ions and solvents molecules were removed for each protein-ligand system, and a distance cutoff of 3.0 was set between points to construct a cluster while leaping every 10th frame.

### 3.7. Cell Proliferative Assay

The 5 × 10^3^ cells/well of MCF-7 or MDA-MB-231cell-lines were seeded in 96 well plates. The next day, cells were treated with 0.16, 0.8, 4, and 20 µM of NSC **13030**, **24198**, **57774**, **137420**, or DMSO (Sigma-Aldrich–Cat D9170, St. Louis, MO, USA) as control. After 48 h of incubation, cells were stained with DAPI (Thermo Fischer–Cat R37606, Waltham, MA, USA) and images of the entire wells were taken with a 20× objective and a DAPI filter cube using Lionheart FX automated microscope. The image analysis and cell counts were done using Gen5 software. All conditions were done in triplicates.

### 3.8. Cell Viability Assay

The percentage of viable cells was determined using The CellTiter 96^®^ AQueous One Solution Cell Proliferation Assay (Promega–Cat G3580, Madison, WI, USA). Briefly, 5 × 103 cells/well of MCF-7 or MDA-MB-231 were plated in a 96 well culture plate in a total volume of 100 µL/well. The next day, cells were treated with 0.16, 0.8, 4, and 20 µM of NSC **13030**, **24198**, **57774**, **137420**, or DMSO as control. After 48 h, 20 µL of CellTiter 96 AQuoeus one solution reagent was added in each well, and incubated for 4 h in a CO_2_ incubator at 37 °C. The percentage of living cells was calculated by dividing the OD (490 nm) of treated cells by the same concentration of DMSO controls. All conditions were done in triplicates.

### 3.9. Enzymatic SHP2 Inhibition Assay

Phosphatase Activity Assay (PTP) using p-nitrophenyl phosphate (pNPP) as a substrate was carried out in a total volume of 100 µL. This was done by monitoring the increase in phosphatase absorbance at 405 nM in the presence of the substrate using 96-well microplate reader (Thermo Scientific Multiskan GO, Waltham, MA, USA). The standard reaction mixture consisted of PTP buffer (100 mM NaCl, 2 mM EDTA, 50 mM HEPES, 3 mM DTT, pH 7.4), 100 nM of Shp2 (BPS Bioscience, Inc., San Diego, CA, USA), and 1 mM of pNPP incubated for 90 min at 30 °C. The enzymatic reaction was stopped by the addition of 50 µL of 5 M NaOH. For inhibition reaction, SHP2 enzyme was preincubated with 100 µM compound or their vehicle (DMSO) for 5 min at 37 °C, prior to the incubation with pNPP substrate for 90 min at 30 °C. The same method was used to calculate IC50 values using GraphPad Prism software 8.0 (San Diego, CA, USA). Wells with SHP2 substrate and without compounds were considered controls (enzyme activity is 100%).

### 3.10. Statistical Analysis

Experiments were carried out in triplicates, three independent times, and were analyzed by two-tailed Student’s *t*-test using Statistical Package for Social Science (SPSS) version 26 (IBM 165 Corporation, Armonk, NY, USA). A *p* value of < 0.05 was considered to be statistically significant.

## 4. Conclusions

The search for novel therapeutic approaches for aggressive tumors is an actual challenge and the identification of new molecular targets is an urgent priority. In this study, we discovered novel SHP2 inhibitors for breast cancer development and progression.

A validated structure-based virtual screening was carried out against the SHP2 catalytic pocket. Consequently, a total number of 35 hits were selected for experimental testing, three compounds, NSC **13030**, **24198**, and **57774**, were found to fit nicely in the SHP2 active site, with a very stable binding mode and to inhibit SHP2 in a selective manner. These three hits significantly decreased the viability and proliferation of the estrogen positive breast cancer, MCF-7. Furthermore, the compound **57774** was found to dramatically decrease the proliferation and viability of the highly aggressive triple-negative breast cancer. MDA-MB-231. The latter compound was found to have a very stable binding mode in SHP2, with a very promising fitting and interaction profile with the active site. This was in line with its in-vitro enzymatic inhibition showing complete SHP2 inhibition at 100 μM, and its IC50 value in the sub-micromolar level (0.8 μM). To our knowledge, these acridine and phenoxazine-based compounds have been tipped for the first time to have anticancer activity through a dual effect, acting as SHP2 inhibitors and potentially as intercalating agents. Therefore, a lead optimization for this compound should assist us and other researchers in the future in converting them into clinically useful anticancer agent.

## Figures and Tables

**Table 1:** The selectivity and specificity of the best performing pharmacophore for active site SHP2 inhibitors.

PH4 No.	Features	Selectivity Se (%)	Specificity Sp (%)
**4**	Aro Acc2 Acc Acc	100	90
**7**	Aro Aro Acc2 Acc	93.2	93.3
**22**	Aro Hyd Acc2 Acc	93.18	66.67
**30**	Hyd Hyd Acc Acc	84.09	80
**39**	Hyd Acc2 Acc Acc	97.72	56.66
**42**	Aro Hyd Acc2 Acc	95.45	60

Aro: Aromatic center, Don: H-bond donor, Hyd: hydrophobic centroid, Acc: H-bond acceptor, Acc2: H-bond acceptor projection, and Don2: H-bond donor projection.

**Table 2:** Shows %Enrichment at various portions of the top-ranked library that was docked into the SHP2 active site using standard protocol (SVS) vs. filter-based protocol (FS).

Top Ranked Portions of the Docked Ligand Library	% Enrichment (% EF)	
	SVS	FVS
1%	4.5	4.5
3%	6.8	13.6
5%	13.6	18.2
10%	20.5	52.3
20%	40.9	95.5

**Table 3:** IC50 values of compounds NSC **13030**, **24198**, **57774**, **137420**, and the control, suramin, on the activity of SHP2 and SHP1. NT: Not tested.

Compound ID	Chemical Structures	SHP2	SHP1
		Obtained IC_50_ (μM)	
Suramin	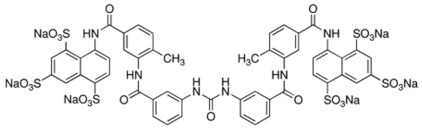	4.5	15.4
**13030**	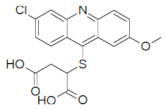	3.2	85.4
**24198**	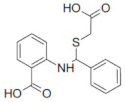	1.9	14.3
**57774**	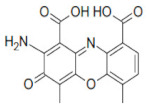	0.8	164.4
**137420**	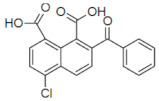	33	NT

**Table 4:** Shows the various pharmacokinetic, druglike, and leadlike properties of our best compounds, as predicted by SWISSADME [33].

		Compound ID			
		13030	24198	57774	137420
Pharmacokinetics	GI absorption	High	High	Low	High
	BBB permeant	No	No	No	No
	CYP1A2 inhibitor	Yes	No	No	No
	CYP2C19 inhibitor	Yes	No	No	No
	CYP2C9 inhibitor	Yes	Yes	No	No
	CYP2D6 inhibitor	No	No	No	No
	CYP3A4 inhibitor	No	No	No	No
	Bioavailability score	0.56	0.56	0.56	0.56
Druglikeness	Lipinski’s rules	Yes	Yes	Yes	Yes
	Veber’s rules	Yes	Yes	No; TPSA >140	Yes
	Ghose’s rules	Yes	Yes	Yes	Yes
	Egan’s rules	Yes	Yes	No; TPSA > 131.6	Yes
	Muegge’s rules	Yes	Yes	Yes	Yes
Leadlikeness	PAINS	No alert	No alert	No alert	No alert
	Brenk’s rules	1 alert; (polycyclic_aromatichydrocarbon_2	1 alert; (het_C_het_not_in_ring)	2 alerts; (aniline, polycyclic_aromatic_hydrocarbon_2)	No alert

## Data Availability

Not applicable.

## Supplementary Materials

The following supporting information can be downloaded at: https://www.mdpi.com/article/10.3390/ijms23084468/s1.
