# Supplementary figures and images for: The Discovery of Potent SHP2 Inhibitors with Anti-Proliferative Activity in Breast Cancer Cell Lines
Source: Int J Mol Sci. 2022 Apr 18;23(8):4468. doi: 10.3390/ijms23084468 (PMC9030381; doi:10.3390/ijms23084468)

## Supplementary Information

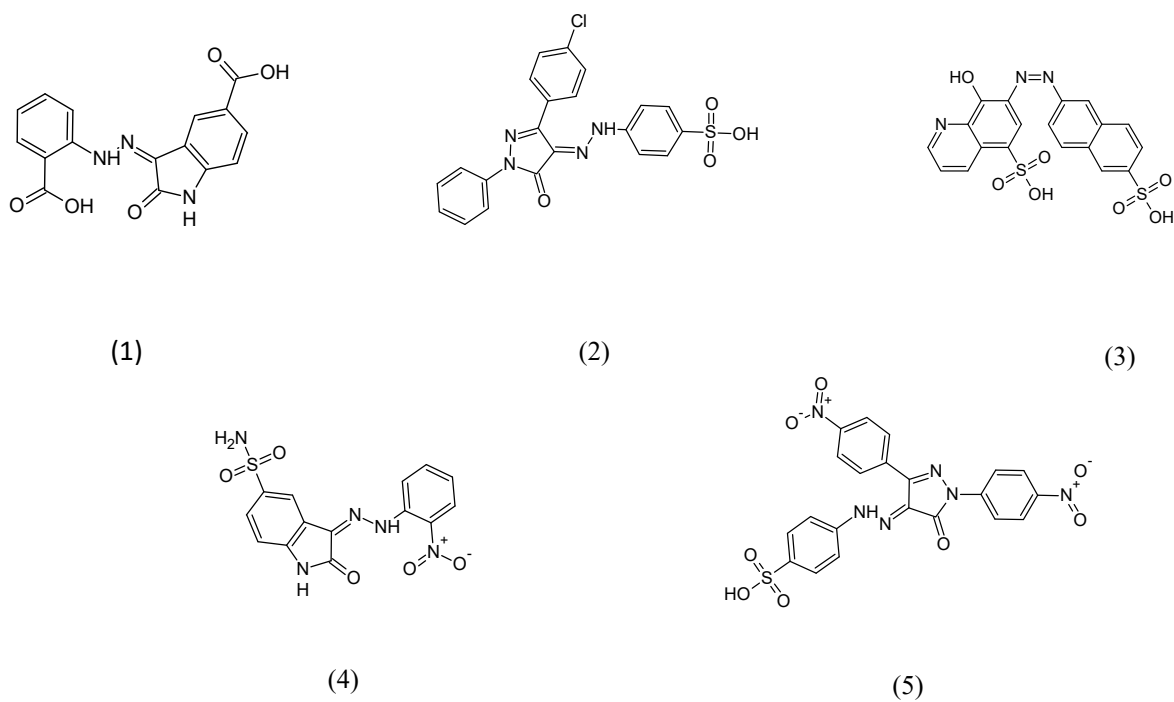

**Scheme 1.** Structure of 14a-isatin (1), PHBS\_3 (2), NSC-87877 (3), 10c-isatin (4) and PHBS\_25 (5).

Supplement: Supplementary file 1 [file ijms-23-04468-s001.zip › ijms-1668193-supplementary.pdf]
